# Prolonged rote learning produces delayed memory facilitation and metabolic changes in the hippocampus of the ageing human brain

**DOI:** 10.1186/1471-2202-10-136

**Published:** 2009-11-20

**Authors:** Richard AP Roche, Sinéad L Mullally, Jonathan P McNulty, Judy Hayden, Paul Brennan, Colin P Doherty, Mary Fitzsimons, Deirdre McMackin, Julie Prendergast, Sunita Sukumaran, Maeve A Mangaoang, Ian H Robertson, Shane M O'Mara

**Affiliations:** 1 School of Psychology & Trinity College Institute of Neuroscience, University of Dublin, Trinity College, Dublin 2, Ireland; 2 Dept of Psychology, National University of Ireland, Maynooth, Co. Kildare, Ireland; 3 School of Medicine & Medical Science, University College Dublin, Dublin 4, Ireland; 4 Dept of Radiology & Diagnostic Imaging, Beaumont Hospital, Dublin 9, Ireland; 5 St Patrick's Hospital, PO Box 136, James's St, Dublin 8, Ireland

## Abstract

**Background:**

Repeated rehearsal is one method by which verbal material may be transferred from short- to long-term memory. We hypothesised that extended engagement of memory structures through prolonged rehearsal would result in enhanced efficacy of recall and also of brain structures implicated in new learning. Twenty-four normal participants aged 55-70 (mean = 60.1) engaged in six weeks of rote learning, during which they learned 500 words per week every week (prose, poetry etc.). An extensive battery of memory tests was administered on three occasions, each six weeks apart. In addition, proton magnetic resonance spectroscopy (^1^H-MRS) was used to measure metabolite levels in seven voxels of interest (VOIs) (including hippocampus) before and after learning.

**Results:**

Results indicate a facilitation of new learning that was evident six weeks after rote learning ceased. This facilitation occurred for verbal/episodic material only, and was mirrored by a metabolic change in left posterior hippocampus, specifically an increase in NAA/(Cr+Cho) ratio.

**Conclusion:**

Results suggest that repeated activation of memory structures facilitates anamnesis and may promote neuronal plasticity in the ageing brain, and that compliance is a key factor in such facilitation as the effect was confined to those who engaged fully with the training.

## Background

The hippocampal formation is a key structure in episodic and spatial memory in humans. Since the original case study of patient H.M. fifty years ago [1], a vast literature has implicated medial temporal lobe structures in memory in both humans and animals [2-4]. Recent decades have seen a delineation of the functions of left and right hippocampi, with left primarily associated with verbally-mediated episodic memory, while the right hippocampus seems crucial for visuo-spatial information [5]. Two features in particular make the hippocampus a unique structure: it was the first region of the brain in which the phenomenon of long-term potentiation [6] was demonstrated in response to pulsed electrical stimulation. LTP remains the most popular neural model of memory formation, and though it has since been shown in other parts of the cortex (E.g. visual cortex: [7,8]; somatosensory cortex: [9]), the hippocampus is still the area in which it is most readily induced [10]. Secondly, the hippocampus is one of the few areas of the brain in which adult neurogenesis occurs; Eriksson et al. [11] have shown that new cells can grow in the dentate gyrus of the hippocampal formation under certain conditions (see also [12,13]). Taken together, these factors suggest the hippocampus may also be a crucial site of plasticity and growth in the mammalian brain (However, several studies point to the importance of entorhinal cortex (EC) rather than hippocampal volume in age-related memory decline (e.g. [14,15]).

Memory problems are the most common cognitive complaint of the elderly to their physicians [16]. The normal decline in memory performance that accompanies old age is thought to be related to cell loss in the hippocampus and prefrontal cortex, two crucial areas for memory encoding and recall [17,18]. Furthermore, extensive neuronal death is observed in these same areas in Alzheimer's Disease [19] although the relationship between memory and structural volume is less clear in healthy adults [14,15]. Paradoxically, older adults display the greatest variation in memory performance over the lifespan, with some older adults capable of performing as well as those in their thirties, while others show evidence of severe memory impairment on standard tasks (e.g. the California Verbal Learning Test (CVLT): [20]). Converging evidence suggests that one mediating factor in this variation may be levels of mental activity. Snowdon and colleagues have shown, in the widely cited "Nuns Studies" [21-23], that continued mental activity in older adulthood is associated with numerous cognitive and health benefits including lower rates of memory decline and neurodegenerative disorders. The nuns in these studies were found to engage in mental activities such as crossword puzzles, Scrabble, word games and other mental exercises. There is a strong implication that repeated activation of cognitive apparatus may be beneficial for brain health and viability, and may even be prophylactic for neural degeneration.

Other evidence supports the role of repetitive cognitive activity in cortical plasticity in the hippocampus. Maguire et al. [24] showed that experienced London taxi-drivers had larger right hippocampi than matched controls, suggesting that repeated accessing of (right hippocampus-mediated) spatial information over a prolonged period may lead to extensive cell growth. Further, Bremner [25] reported Vietman veterans suffering from post-traumatic stress disorder (PTSD, characterised by repeated re-experiencing of traumatic events) had shrunken hippocampi, with on average an 8% loss of volume. The accompaniment of stress (as measured by increases in blood cortisol) with re-experiencing may have been neurotoxic, resulting in hippocampal shrinkage. When coupled with the large LTP literature on altered neuronal firing following electrical stimulation, this evidence may imply that repeated use of memory processes, with the concomitant repetitive activation of their neural substrates such as the hippocampus, leads to changes in both neural signalling and cell structure. However, the above studies suffer from the serious limitation of being cross-sectional in design, thereby not allowing for any experience-based plastic changes to be tracked over a period of time. The current study employs a longitudinal design wherein any plastic changes may be observed over a period of three months.

Repeated rehearsal of verbal material is termed "rote learning", and is a common method by which small amounts of information can be transferred from short-term to long-term memory (e.g. telephone numbers, email addresses etc.; [26]). Rote rehearsal activates a distributed neural circuit consisting of left inferior prefrontal cortex, supplementary motor area (SMA), bilateral posterior parietal cortex, lateral cerebellum and medial temporal lobe (including hippocampus); furthermore, activity levels in these structures during rehearsal predict subsequent recall of the rehearsed material [27]. Rausch and Babb [28] examined rote-learned word-pairs and hippocampal cell loss in epilepsy patients pre- and post-surgery; they found a significant relationship between degree of cell loss and rote learning in left, but not right, hippocampus. Rote learning might be considered a means by which repetitive activation of particular memory structures (especially the medial temporal lobe, prefrontal cortex and the fibre pathway connecting them, the uncinate fascicle; [29]) can be accomplished.

Here we attempt to enhance memory function in healthy aged participants through the use of prolonged rote learning by repetitively activating memory structures in the brain. We predict that this repetitive activation may effect cortical plasticity (most likely dendritic growth processes) or promote cell health/viability, as indexed by single voxel proton magnetic resonance spectroscopy (^1^H-MRS). Valenzuela et al. [30] have shown that mnemonic memory training using a spatial strategy produced improvements on word-list recall and metabolic changes in the hippocampus. They found training-related alterations in concentrations of creatine (Cr) and choline (Cho), but not N-acetylaspartate (NAA) after five weeks of training using the Method of Loci (MoL) to recall lists of words. These biochemical changes related to cellular energy and cell-membrane metabolism rather than number and health of neurons [31,32]. Such memory-related metabolic change was predicted over 50 years ago: Hebb [33], in stating his theory of synaptic plasticity, referred to "repeated and persistent" activation of synapses leading to "some growth process or metabolic change" taking place such that the efficiency of that cell pairing is increased. In a study of nondemented older adults Zimmerman et al. [34] demonstrated that participants with reduced hippocampal NAA/Cr ratio performed more poorly on a test of verbal memory. Based on these findings they suggested that the integrity of both the structure and metabolism of the hippocampus may underlie verbal memory function. A further study of younger healthy participants found that an NAA increase was associated with overall neuropsychological performance which may be related to mitochondrial function [35]. Huang et al. [36] associated a reduction in NAA with the level of cognitive dysfunction and Riederer et al. [37] found a correlation between increased NAA concentrations in the temporal lobe and verbal memory. Charlton et al. [38] found that reduced NAA, increased Cho and increased Cr were associated with decline in executive function performance/cognitive function consistent with their role of axonal integrity and cellular energy metabolism. They found that beyond the effects of age and estimated intelligence, only NAA significantly contributed to explaining the variance in executive function. This has been supported by Kantarci et al. (2002) and Olson et al. [39,40]. However Valenzuela et al. [30] reported an unexplained reduction in NAA/Cr measures in the hippocampus following memory training. This last finding may be explained by increased neural efficiency resulting from the memory training, as none of the other studies mentioned above employed a specific training régime. As such, while increases in NAA (or NAA/Cr or Cho ratio) may be seen as indices of general memory or cognitive function, decreases in NAA (or derived ratios) might be a more likely indicator of cognitive change following memory training.

We predict postlearning memory enhancement, specifically on verbal memory tasks, accompanied by metabolic changes in memory-related brain structures, specifically prefrontal cortex and/or MTL areas including the hippocampal formation and EC. Specifically, in the event of generalised training effects manifested across all tasks, we would predict decreases in NAA/(Cr+Cho) ratio, consistent with Valenzuela et al [30] above and supporting the claim of increased neural efficiency in a reduced number of cortical circuits. However, performance gains specific to verbal memory tasks (e.g. Rivermead Short Story test, CVLT) should be associated with increases in NAA concentration and/or NAA/(Cr+Cho) ratio, consistent with Zimmerman et al. [34] and Riederer et al. [37]. Finally, any such changes are predicted to be evident in brain regions specifically associated with memory - hippocampus and/or prefrontal cortex - and *not *in the control voxel, placed at midline prefrontal cortex, a region not typically associated with memory function.

## Methods

### Participants

Twenty-four participants (9 male) between the ages of 55 and 70 years (mean = 60.1) were recruited by means of a newspaper advertisement requesting volunteers for a study on memory. Exclusion criteria were any self-reported history of neurological, medical or psychiatric disorder, alcohol or drug addiction, epilepsy, heart problems, serious head injury resulting in loss of consciousness or psychoactive medication. None of the participants reported any known vascular risk factors such as hypertension or diabetes, as such were included in the exclusion criteria. Claustrophobia or the presence of any bodily ferrous metals (pacemakers, aneurism clips, metal pins/plates) excluded participants from the MRS aspect of the study. All structural MRS scans were inspected for abnormalities by a trained radiographer. Participants were paid only for full completion of the study. Written and informed consent were obtained prior to commencement. The experiment conformed to the Declaration of Helsinki and was approved by the Trinity College Psychology Department Ethics Committee and that of Beaumont Hospital, Dublin.

### Design

**Figure 1:**
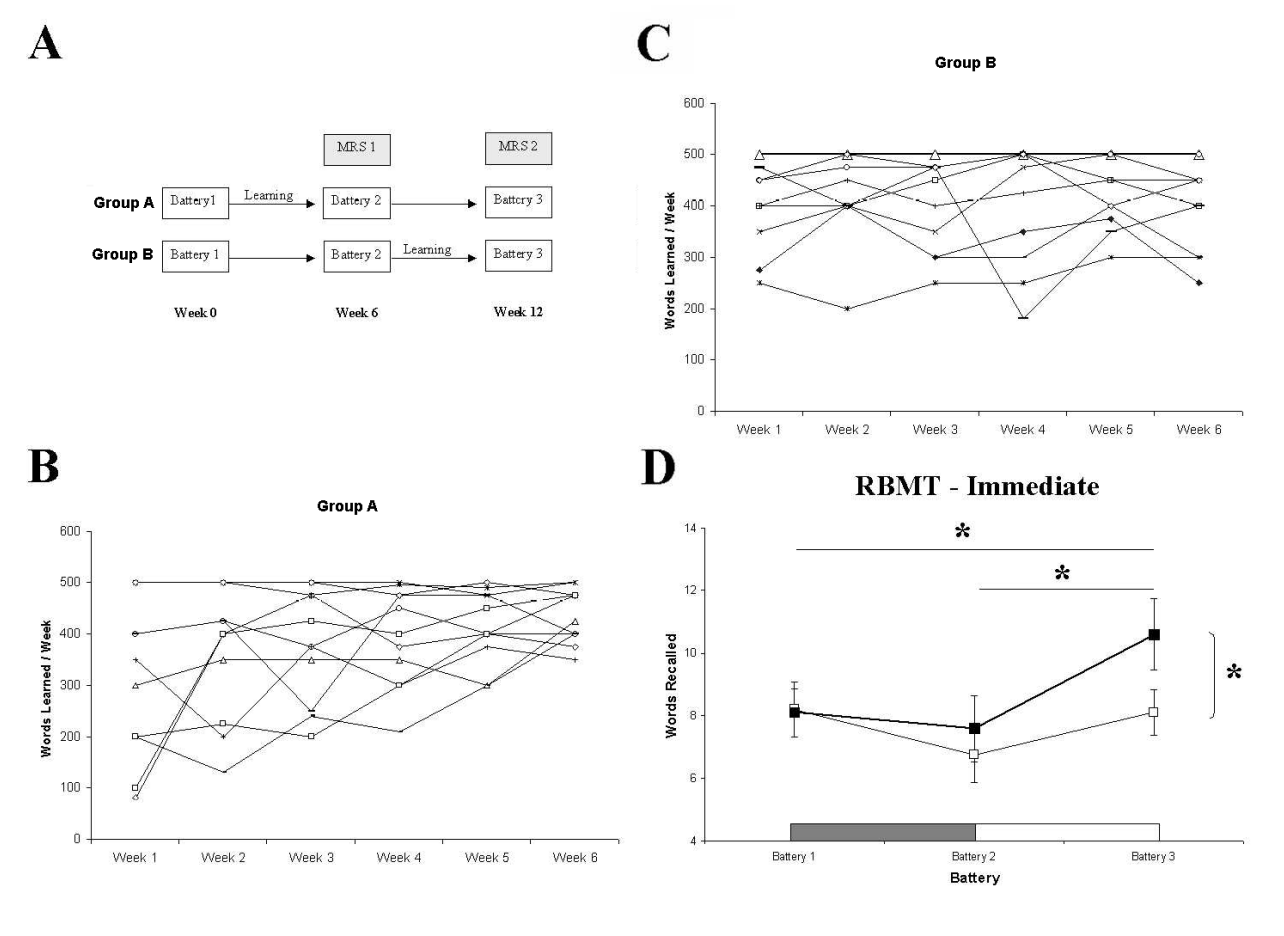
**Experimental design, words learned per week and RBMT-E recall scores for Groups A and B**. A) Schematic of crossover experimental design: Group A learned from Week 1 to Week 6; Group B learned from Week 6 to Week 12. Both groups completed memory test batteries at Week 0, 6 and 12, and all consenting participants were scanned with MRS at Weeks 6 and 12. B&C) Weekly words learned per participant for Group A and Group B (three non-compliant participants removed) demonstrating significantly larger mean improvement across the six weeks for Group A. D) Immediate Recall of the short story subtest of the RBMT. Group A (heavy line, filled squares) showed a significant improvement in recall relative to Group B (thin line, empty squares) at Battery 3, and a significant within-groups improvement relative to Battery 1 and 2. Horizontal bars represent 6-week learning blocks for Group A (grey bar) and Group B (white bar).

Stratified random sampling was carried out to allocate the participants into two groups of twelve, using the strata Gender and Age. The groups were also matched for intelligence, based on NART score (National Adult Reading Test) and absentmindedness, based on CFQ score [41]. A crossover design was used; all participants completed a battery of learning and memory tests at baseline, before learning commenced (Battery 1). Group A then spent the first six weeks engaged in intensive rote learning of verbal material (see below) while Group B remained at baseline. At the end of the six weeks, a second battery was completed by all participants (Battery 2) containing alternate forms of the learning and memory tests. The groups then reversed, with Group B spending six weeks learning while Group A ceased rote rehearsal. At the end of this six week block, a third battery was administered (Battery 3). Six weeks later, at Week 18, a fourth and final battery (Battery 4) was administered to test for any delayed effects in Group B (See Figure 1, A for schematic of design). The addition of Battery 4 allowed for a straight comparison of pre-training, post-training and six weeks post-training to be carried out, thereby allowing delayed effects to be investigated and simultaneously extending beyond the limitations of the standard crossover design.

### Procedure

#### Rote Learning

During their six weeks of rote learning, participants were required to learn 500 words of verbal material per week every week. They were allowed to choose their own material (poetry, prose, song lyrics, newspaper articles etc.) in the hope that this would lead to a high rate of compliance. A selection of material of the appropriate length was also provided for participants who preferred not to find their own material. All but one participant chose to learn the material provided by the experimenters (The material used by this participant was inspected by the experimenters prior to the study and assessed for its content, difficulty and volume, and was judged to be appropriate for the study). Recall of the material was tested every week, and a record kept of the number of words learned each week. No instruction was given as to what learning strategy to use, but it was suggested that the participants learn part of the material each day rather than all in one session by reading over it repeatedly. Participants were asked after their learning whether they had used any particular strategy, and all reported the use of rote repetition.

#### Behavioural Testing

At each of the behavioural batteries, an array of tests of learning and memory (both verbal and visuo-spatial) and executive function were administered. These are described briefly.

Two measures of verbal learning and memory were used. The **Short Story Recall **task of the Rivermead Behavioural Memory Test - Extended version (**RBMT-E**) consists of a short paragraph of prose (5 to 6 lines) which is read aloud to the participant for recall. The passage is divided into 21 ideational units, and each unit correctly recalled scores one point. This task involves both immediate (directly after it is read aloud) and delayed recall (25-30 minutes later), and has 6 alternate forms which are equated for difficulty. The **California Verbal Learning Test (CVLT) **is a word-list learning task. A list of sixteen words is read aloud for immediate recall (the words are organized into four semantic categories). The list is then read again for a second recall trial, and repeated for five recall trials. An interference list is then read, also of sixteen words, for immediate recall. The participant must then recall the original list without hearing it read again. There then follows a cued recall test. After a delay of 25-30 minutes, the participant must again recall as many words from the original list as possible. Cued recall and a yes/no recognition task then follow. The CVLT provides indices of short- and long-term free and cued recall, as well as learning slope, clustering, proactive and retroactive interference.

A **Visuo-spatial Learning task (VSL) **was also used. Participants were presented with a 10 × 10 cell grid pattern consisting of eleven shapes (filled in blue squares) against yellow empty squares. The grid was presented on screen for 2 minutes, during which participants were instructed to study the shapes. They were then presented with an empty grid on a response sheet, and instructed to reproduce the pattern by filling in the blue squares with pen. Four versions of this task were used, equated for the number of shapes. Scoring yielded a total VSL score, visual score (number of shapes recalled) and spatial score (number of locations correctly recalled).

A measure of episodic memory for everyday events, the **Mundane Memory Questionnaire (MMQ) **was also given. The MMQ is a pen and paper measure of recall of twelve commonplace everyday events over the last four days (E.g. "Do you recall getting dressed on this day? If so, what did you wear?"). One point is scored for responding "yes" and a second point given for supplying details. A score is obtained for each of the four past days. This test has been shown to reliably differentiate hippocampectomied patients from normals (Mangaoang M, McMackin D, Fitzsimons M, Delanty N, Phillips J, Quigley J, O'Mara SM: Unilateral hippocampal damage impairs spoken and written language, Submitted). A sustained attention task, the **SART (Sustained Attention to Response Task**; [42]), was also given. The SART requires participants to make a button-press response to a stream of rapidly-presented random digits (1-9) with the task rule that response must be withheld for the number 3. This task is known to be subserved by the right hemisphere prefrontal-parietal attention network [43] and is a measure of frontal executive function and attention.

In addition to the NART and the CFQ, three other control measures were administered: the **Hospital Anxiety and Depression Scale (HADS) **was given at each battery to monitor levels of depression and anxiety throughout the study. In addition, the **Perceived Stress Inventory **[44] was also included, given the known deleterious effects of stress on learning (Mullally SL, Brotons JR, Cowley TR, Gobbo O, Shaw KN, O'Dwyer AM, O'Mara SM: Combined, but not separate, physical exercise and fluoxetine treatment ameliorates the effect of repeated moderate stress on hippocampal-dependent spatial learning, Submitted). In the light of recent findings regarding exercise and learning, an **Exercise Questionnaire **(adapted from [45]) was also given to all participants at the end of the study.

Behavioural batteries lasted approximately 90 minutes, and were split into two sessions with a ten-minute break between sessions. Tests of verbal learning and memory were divided between the sessions to control for any possible interference effects of list content, and it was made clear at the start of the second session that no material from the previous session would be requested.

### Magnetic Resonance Spectroscopy

In addition to behavioural testing, participants who were suitable and willing for MR scanning (Group A, n = 9, Group B, n = 6) underwent proton spectroscopy (^1^H-MRS) at Week 6 (immediately post-learning for Group A and pre-learning for Group B) and Week 12 of the study (six weeks after learning ceased for Group A and immediately post-learning for Group B).

Single voxel ^1^H MRS studies were conducted using PROBE software on a GE Signa 1.5 Tesla system (GE, Milwaukee, WI, USA). Subjects were positioned in the supine position so that the centering lights passed along their median sagittal plane and along their inter-pupilary line. Distance measurements were taken from the outer canthus of each eye to the base of the headrest to ensure both sides were equidistant. A protractor was then used to measure the angle between the subject's anthropomorphic baseline and the vertical. Voxels of interest (VOIs) were located on a set of coronal T2- weighted images acquired from the anterior-most portion of the brain to the posterior aspect of the lateral ventricles. Metabolite levels of N-acetylaspartate (NAA), creatine + phosphocreatine (tCr) and choline (Cho) were obtained from seven VOI locations: right and left prefrontal cortex, midline prefrontal, and right and left anterior and posterior hippocampus. VOIs measured 2 cmx2 cmx2 cm (8 cm^3^) each. Precise positioning of VOIs at first and repeat MRS acquisitions was determined by distance measurement from anatomical markers visible on the T_2 _image set. Spectra were acquired using point-resolved spectroscopy (PRESS; TR, 1500; TE, 35).

Using the automated shimming methods available on the GE Signa system, two approaches were employed to correct for field inhomogeneity as previously described by Kegeles et al. [46]. Phase differences between two echoes of different echo time are used to adapt shim currents to correct for inhomogeneity using the phase map method. Voxel shimming which calculates the optimal shim current to reduce the line width of water and correct for inhomogeneity was the second method employed. The latter is unique to magnetic resonance spectroscopy sequences. In the current study such techniques were essential due to the location of hippocampal voxels in the temporal lobe in close proximity to areas of potential inhomogeneity, the skull base and sphenoid sinus

As highlighted by Kreis [47] and Taylor [48] quality checks on MRS data are essential. Thus for each examination the location of VOIs was checked pre- and post-MRS, individual raw data files were stored to facilitate checks for motion, system instabilities and SNR, water reference scans were used to correct for eddy currents using SAGE (Spectroscopy Analysis of GE, GE Healthcare, Milwaukee, WI, USA) using the Probe/SVQ automatic single voxel processing routine, spectral resolution was improved by using a dual shimming technique, and spectra were assessed for abnormalities (failure of water saturation, ghosts, peak doubling, unidentified metabolites, and signal from outer volume). Spatial saturation bands were also positioned to completely surround the VOI.

After acquisition the Probe/SVQ software conducts an automatic analysis. Using the phase corrected water reference signal and the phase corrected suppressed signal, a pure metabolite signal is computed through a water subtraction technique. A narrow frequency window around each of the metabolite resonances is analyzed by curve fitting, using the Marquardt-Levenberg method. Before curve fitting, the spectral line shape is manipulated to improve fitting by removing any errors associated with line width variations. There are three line shape manipulations: line broadening, line width normalization, and line shape transformation. Each of these manipulations are combined into a single apodization step. All Probe/SVQ numerical analysis is based on peak amplitude but by normalizing the line widths of the peaks, the analysis effectively measures areas and ratios of areas. The algorithm first determines the line width of the creatine peak, a (partial) Lorentzian to Gaussian transformation is performed, the line width normalization and the first part of the line shape transformation are combined into a single exponential apodization, and the second half of the line shape transformation is performed by a Gaussian apodization.

Signal intensities of the metabolite peaks are determined from which the ratio NAA/(tCr+Cho) is established. Producing an image containing a Pure Absorption (Real Part FFT) Spectrum covering a spectral range from -0.4 to 4.3 parts per million (ppm), and an associated chemical shift scale axis with tick marks in ppm.

### Data Analysis

Behavioural data were analysed using mixed factorial ANOVAs with within-groups factor BATTERY (Battery 1 - Battery 3) for crossover analyses or PRE-POST (Pre-training, Post-training and 6 Week delay) for delayed effects analysis, and between groups factor GROUP (Group A, Group B). The DV in each analysis was the dependent measure obtained from each behavioural test. Lack of sphericity was compensated for, where appropriate, using a Greenhouse-Geisser correction (indicated by "GG" after the p-value). Within-groups main effects were explored using Bonferroni multiple comparisons. All means are reported ± SEM; due to the large number of analyses, only significant F and p-values for main effects/interactions are reported. Pearson correlations were carried out between behavioural performance measures and Total Words Learned (TWL) for each participant, as well as the increase in words learned from Week 1 to Week 6 ("delta words", or words).

Spectroscopy data were analysed by computing the ratio of NAA to the sum of creatine and choline (i.e. NAA/(tCr+Cho)); this ratio was used because creatine and choline are considered relatively stable metabolites, thereby allowing changes in NAA to be highlighted. This ratio was computed at each of the seven VOIs at Week 6 and Week 12. A series of mixed factorial ANOVAs were carried out for the seven voxels of interest with GROUP (2 levels) as the between groups factor and PRE-POST (2 levels) as the within groups factor; NAA/(tCr+Cho) ratio was the DV in each case. Greenhouse-Geisser and Bonferroni corrections were again employed, where appropriate. Pearson correlations were also computed between the change in metabolite ratios and behavioural measures of memory performance.

## Results

### Control Measures

Table 1 shows a summary of results for the control measures. The groups did not differ significantly on mean NART, Exercise or CFQ scores (NART: Group A = 30.7 ± 3.2, Group B = 36.8 ± 2.6; Exercise: Group A = 70.7 ± 4.27, Group B = 66.5 ± 7.9; CFQ: Group A = 40.5 ± 3.8, Group B = 44.0 ± 3.0) as measured at Week 0/Battery 1. No main effects of GROUP, BATTERY or GROUP × BATTERY interactions were found for the HADS (Total, Depression & Anxiety), Perceived Stress Inventory or SART (Commission Errors/Correct Withholds). A significant main effect of BATTERY was found for SART Omission Errors; Bonferroni tests revealed a significant reduction in omission errors from Battery 1 to Battery 3 (9.5 ± 2.3 vs. 3.2 ± 0.7; t = 2.87, df = 20, p = 0.027).

**Table 1:** Control Measures for Group A vs. Group B across Batteries 1-3.

Measure	Main Effect BATTERY	Main Effect GROUP	Interaction BATERY × GROUP
NART Score	**-**	t(19) = 1.48, p = .16	**-**
CFQ	**-**	t(19) = 0.73, p = .48	**-**
HADS Total	F(1,19) = 0.12, p = .89	F(1,19) = 0.6, p = .45	F(1,19) = 0.6, p = .55
HADS Depression	F(1,19) = 0.49, p = .57 GG	F(1,19) = 3.4, p = .08	F(1,19) = 0.15, p = .86
HADS Anxiety	F(1,19) = 0.41, p = .67	F(1,19) = 0.02, p = .88	F(1,19) = 0.96, p = .39
PSI Score	F(1,19) = 0.48, p = .62	F(1,19) = 0.42, p = .53	F(1,19) = 0.09, p = .92
SART Commission Errors	(1,18) = 2.9, p = .07 GG	F(1,18) = 0.01, p = .94	F(1,18) = 0.23, p = .8
SART Omission Errors	F(1,18) = 3.9, p = .045 GG*	F(1,18) = 2.0, p = .18	F(1,18) = 0.09, p = .92

Key: Significant main effects are asterisked (GG signifies Greenhouse-Geisser corrected significance).

### Rote Learning

Three participants were removed from the analysis because their total words learned fell below 1,500 (mean <250 words/week, implying <50% compliance). Compliance with weekly rote learning varied across the two groups; Group A exhibited a high degree of compliance with weekly learning, while Group B did not. While the two groups were almost identical for Total Words Learned (TWL) over the six-week training block (Group A = 26,671; Group B = 26,405), the mean increase in words learned from Week 1 to Week 6 was 113.2 (± 44.6) words for Group A, and 10.0 (± 16.7) words for Group B, indicating a significantly greater performance enhancement and greater compliance in Group A (t = 2.17, df = 19, p = 0.05; Figure 1, B &1C).

### Behavioural Testing

Memory facilitation was investigated using Batteries 1-3 in the crossover element of the design. The groups were compared at Batteries 1, 2 and 3 to test for behavioural improvement on memory tasks immediately after six weeks of rote learning (i.e. at Battery 2 for Group A and Battery 3 for Group B). Data from the Visuo-Spatial Learning (VSL) task were translated into z-scores to correct for differing difficulty levels across batteries. Immediate recall on the VSL revealed no significant effects for total VSL score, visual score or spatial score. Similarly, no significant differences were found for delayed recall on total score, visual score or spatial score.

Immediate recall on the short story task from the Rivermead Behavioural Memory Test revealed a significant main effect of BATTERY (F(2,38) = 5.15, p = 0.01) but not of GROUP (F(2,38) = 1.03, p = 0.32) or GROUP × BATTERY (F(2,38) = 1.88, p = 0.17). This main effect was driven by a difference across batteries in Group A (F(2,20) = 6.05, p = 0.009). Bonferroni multiple comparisons showed that for Group A, immediate recall on Battery 3 was significantly higher than for Battery 1 (t = 3.5, df = 10, p = 0.018) and Battery 2 (t = 2.8, df = 10, p = 0.051); mean recall for Battery 1 was 8.09 units compared to 7.59 units for Battery 2 and 10.59 units for Battery 3 (see Figure 1, D). Delayed recall showed no significant differences for any comparison (BATTERY: F(2,38) = 2.07, p = 0.14; GROUP: F(2,38) = 2.21, p = 0.15; BATTERY × GROUP: F(2,38) = 1.3, p = 0.28).

**Figure 2:**
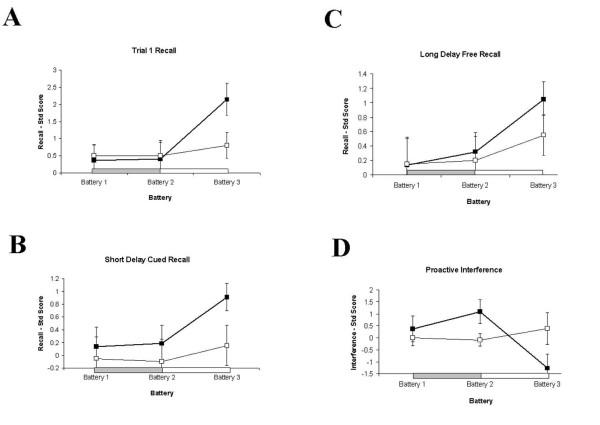
**Behavioural data showing recall on four CVLT subscales across batteries and groups**. Four selected subscales of the CVLT demonstrating delayed six-week memory facilitation for Group A (heavy line, filled squares). Horizontal bars represent 6-week learning blocks for Group A (grey bar) and Group B (white bar). A) Word recall on Trial 1, showing significant increase in recall for ote A relative to Group B (thin line, empty squares) at Battery 3. B) Short Delay Cued Recall for Group A and Group B, also exhibiting significant improvement at Battery 3. C) Long Delay Free Recall for Group A and Group B, showing the same pattern of delayed facilitation at Battery 3. D) Proactive Interference for the two groups, showing a significant decrease in interference for Group A at Battery 3. This pattern of delayed enhancement of memory was also found for Trial 5, Total Trials 1-5, Semantic Clustering and Subjective Clustering (not shown).

On the CVLT, eight separate measures revealed a pattern of improved memory scores on Battery 3 relative to Batteries 1 and 2, for Group A (Figure 2 shows four representative effects). These measures were Recall on Trial 1, Recall on Trial 5, Total Recall for Trials 1-5, Short Delay Cued Recall (SDCR), Long Delay Free Recall (LDFR), Semantic Clustering, Subjective Clustering and Proactive Interference (lower levels of proactive interference were observed for Battery 3 in Group A). The effects on these measures could not be attributed to increasing task familiarity, as the improvement was absent for Group B. Repeated-measures ANOVA analysis showed that these main effects were driven by differences between Battery 2 and Battery 3, suggesting a delayed effect of rote training for Group A. Across-battery differences for both groups were evident on four other measures (Short Delay Free Recall, Across Trial Consistency, Cued Recall Intrusions and Total Recognition), indicating that these improvements were due to familiarity with the task/testing procedure.

**Figure 3:**
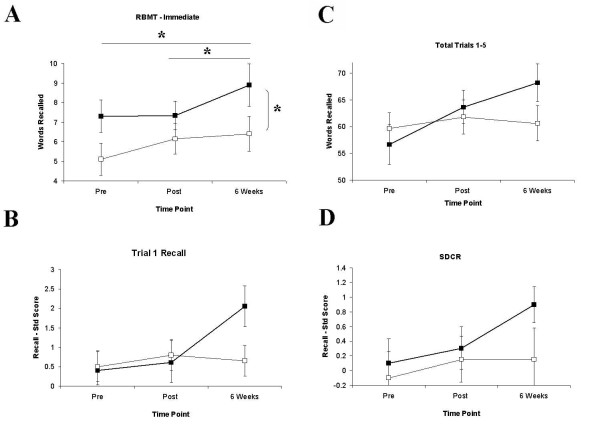
**Behavioural data showing recall on RBMT-E and CVLT measures at Pre-, Post- and 6 Weeks Post-training**. A) Pre, Post and 6 Weeks Post immediate recall for RBMT showing significant within-groups main effect for Group A (heavy line, filled squares) and between-groups difference at 6 Weeks Post. B-D) Delayed recall facilitation for Group A (heavy line, filled squares) but not Group B (thin line, empty squares) on CVLT sub-measures Trial 1 recall, Total Recall Trials 1-5 and Short Delay Cued Recall (SDCR); this pattern was also shown for Trial 5 recall and Long Delay Free Recall (LDFR; not shown).

**Figure 4:**
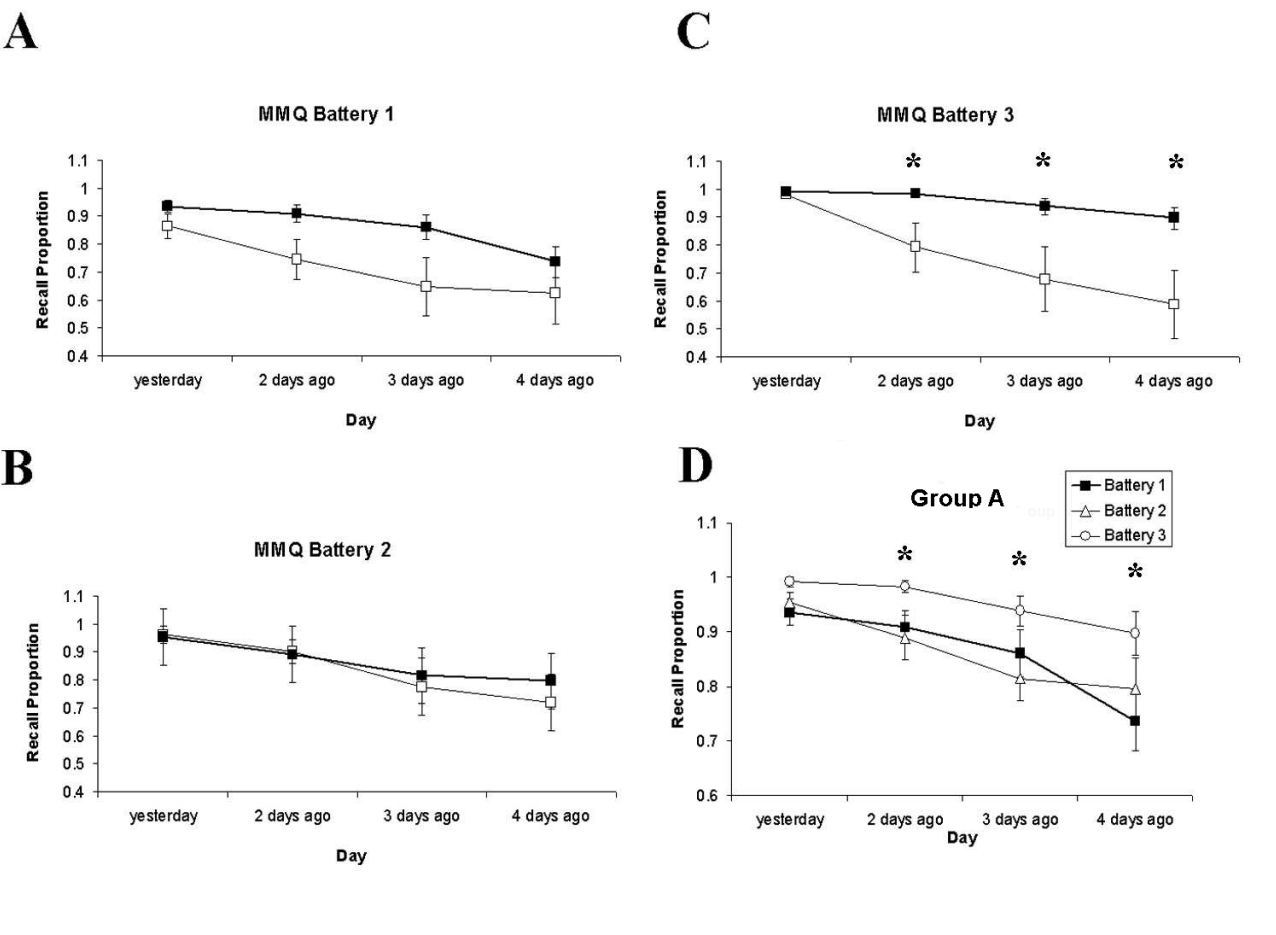
**Behavioural data showing recall on the Mundane Memory Questionnaire across batteries and groups**. Mundane Memory Questionnaire recall scores for Group A (heavy line, filled squares) and Group B (thin line, empty squares) for Battery 1 (A), Battery 2 (B) and Battery 3 (C). Differences between groups were significant at Battery 3 for information from 2, 3 and 4 days ago, with superior recall in Group A. D) Within-groups recall increase for Group A across Batteries 1-3, showing improved recall for daily events at Battery 3 relative to Battery 2 at 2 and 3 days ago, and for Battery 3 relative to Battery 1 for 4 days ago.

**Figure 5:**
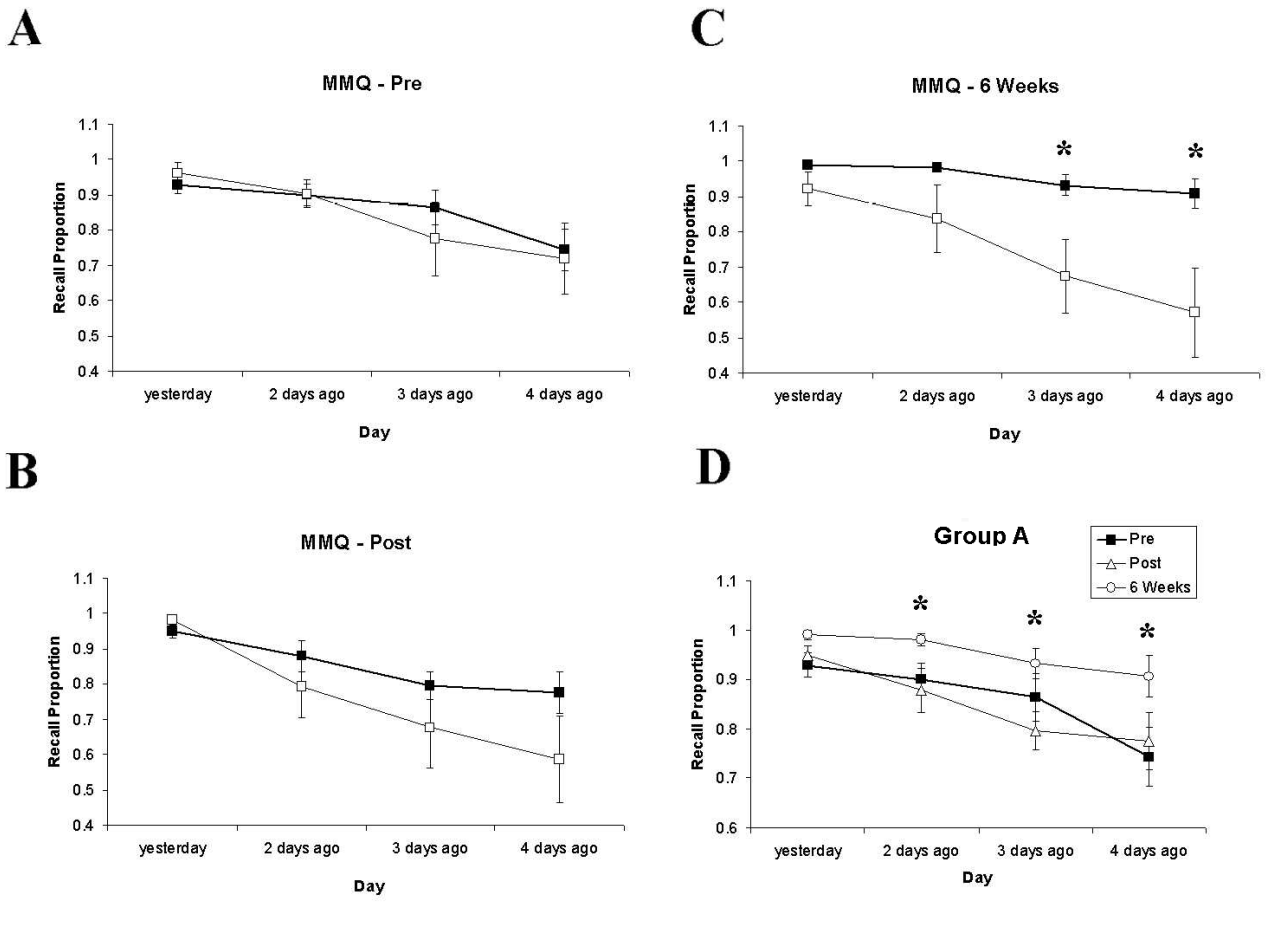
**Behavioural data showing recall on the Mundane Memory Questionnaire at Pre-, Post- and 6 Weeks Post-training**. MMQ recall scores for Group A (heavy line, filled squares) and Group B (thin line, empty squares) at Pre (A), Post (B) and 6 Weeks Post (C). Differences between groups were significant at 6 Weeks Post for information from 3 and 4 Days Ago, with superior recall in Group A. D) Within-groups recall increase for Group A across time points, showing improved recall for daily events at 6 Weeks Post relative to Post at 2 and 3 Days Ago, and for 6 Weeks relative to Pre for 2 and 4 Days Ago.

The Mundane Memory Questionnaire (MMQ) also revealed a delayed memory enhancement for Group A (Main Effect of GROUP: F(1,19) = 4.15, p = 0.056; Figure 3, A-C). While Main Effects of DAY were evident overall (F(3,57) = 18.63, p = 0.001, GG) and at each battery individually (Battery 1: F(3,57) = 11.22, p = 0.001, GG; Battery 2: F(3,57) = 7.69, p = 0.002, GG; Battery 3: F(3,57) = 10.07, p = 0.001, GG), a Main Effect of GROUP (F(3,57) = 6.67, p = 0.018) and a DAY × GROUP interaction were found at Battery 3 only (F(3,57) = 3.9, p = 0.024, GG). Group A showed a significant enhancement at Battery 3 for events occurring two days (t = 2.16, df = 19, p = 0.03), three days (t = 2.19, df = 19, p = 0.027) and four days previously (t = 2.41, df = 19, p = 0.018). Within-groups differences were also found for Group A from Battery 2 to Battery 3 for two days ago (t = 2.7, df = 10, p = 0.022) and three days ago (t = 4.28, df = 10, p = 0.002). For events occurring four days previously, Group A showed a significant enhancement in recall from Battery 1 to Battery 3 (t = 2.49, df = 10, p = 0.032; Figure 3, D). No significant differences on any of these comparisons were found for Group B (see Figures 4 and 5)

### Magnetic Resonance Spectroscopy

**Figure 6:**
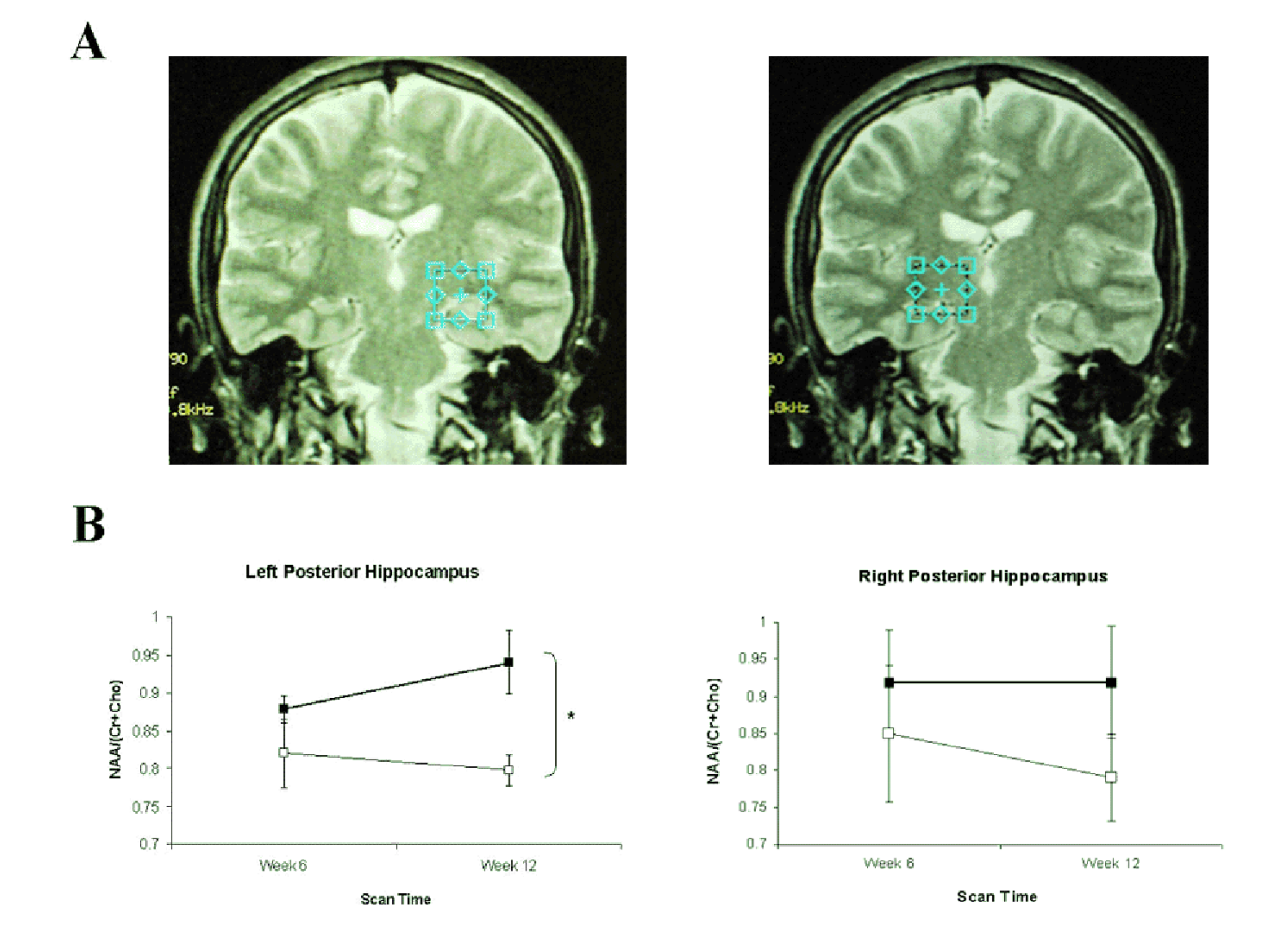
**MR Spectroscopy voxel location, metabolic measures across batteries and schematic representing correlations between behavioural performance and metabolism**. A) Localisation of the left and right posterior hippocampal voxels (2 cm × 2 cm × 2 cm) used in MRS scanning. B) MRS metabolism as measured by ratio of NAA/(tCr+Cho) at the left and right posterior hippocampal voxels. At Week 12/Scan 2, Group A (heavy line, filled squares) had a significantly larger ratio than Group B (thin line, empty squares) for the left posterior hippocampus; no significant change was observed at the right posterior hippocampal voxel.

**Figure 7:**
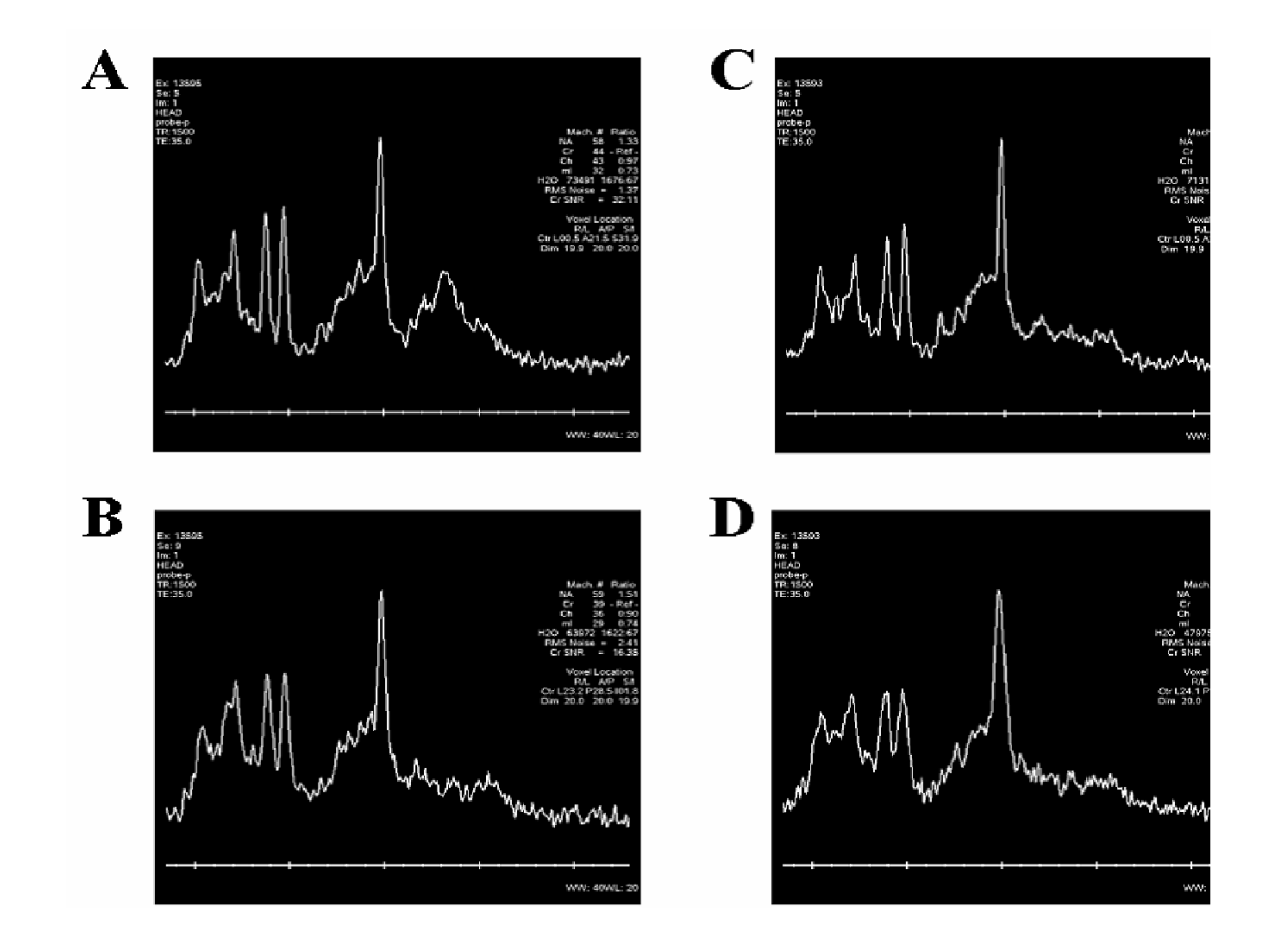
**Representative spectra from two selected participants (#1 and #2) showing Voxels of Interest (VOIs) at unaffected Midline Prefrontal and affected Left Hippocampal regions**. A) Representative spectrum from the non-affected midline prefrontal VOI of Participant #1. B) Representative spectrum from the left posterior hippocampus VOI of Participant #1 showing an increase in NAA/(tCr+Cho) after prolonged rote learning. C) Representative spectrum from the non-affected midline prefrontal VOI of Participant #2. D) Representative spectrum from the left posterior hippocampus VOI of Participant #2 showing a significant increase in NAA/(tCr+Cho) after prolonged rote learning.

A significant main effect of GROUP was found at one voxel of interest (VOI), left posterior hippocampus (F(1,13) = 9.13, p = 0.01) for the ratio of NAA/(tCr+Cho). At Week 12/Scan 2, Group A had a significantly larger ratio than Group B (Group A: 0.94 ± 0.04, Group B: 0.79 ± 0.05; t = 2.62, df = 13, p = 0.021; Figure 6, A &6B). No other VOIs revealed significant main effects or interactions (see Figure 7A-D for representative spectra from two participants). Complete MRS ratios (NAA/Cr, NAA/Cho, Cho/Cr) for each voxel in each group are shown in Table 2.

**Table 2:** MRS ratios of NAA/Cr, NAA/Cho and Cho/Cr for Group A and Group B at each Voxel of Interest (VOI) at Week 6 and Week 12.

		**NAA/Cr**		**NAA/Cho**		**Cho/Cr**	
	
	**VOI**	**Week6**	**Week12**	**Week6**	**Week12**	**Week6**	**Week12**
	
**Group A**	**Right PFC**	**1.49** (1.41,1.6) *1.33,1.72*	**1.56** (1.44,1.63) *1.24,1.7*	**0.96** (0.83,1.08) *0.77,1.16*	**0.87** (0.8,0.93) *0.76,1.11*	**1.56** (1.39,1.74) *1.22,1.99*	**1.73** (1.47,1.92) *1.33,2.04*
	**Left PFC**	**1.64** (1.5, 1.71) *1.43,1.87*	**1.60** (1.43,1.71) *1.3,1.79*	**0.96** (0.92,1.04) *0.76,1.13*	**0.87** (0.84,1.04) *0.71,1.25*	**1.69** (1.55,1.91) *1.43,1.99*	**1.61** (1.46,1.94) *1.43,2.25*
	**Midline PFC**	**1.51** (1.46,1.58) *1.38,1.74*	**1.53** (1.4,1.57) *1.29,1.76*	**0.82** (0.77,0.95) *0.75,1.35*	**0.91** (0.73,1.00) *0.68,1.18*	**1.74** (1.61,1.93) *1.29,2.11*	**1.67** (1.51,2.04) *1.3,2.13*
	**Right Ant. Hipp**.	**1.66** (1.44,1.81) *1.22,2.03*	**1.56** (1.43,1.62) *1.19,1.84*	**1.04** (0.93,1.16) *0.87,1.22*	**1.04** (0.97,1.09) *0.88,1.12*	**1.69** (1.38,1.82) *1.0,2.04*	**1.48** (1.4,1.66) *1.29,1.82*
	**Left Ant. Hipp**.	**1.63** (1.46,1.7) *1.41,1.94*	**1.70** (1.54,1.8) *1.4,1.93*	**0.97** (0.92,1.11) *0.83,1.29*	**1.07** (1.0,1.16) *0.82,1.3*	**1.72** (1.47,1.79) *1.16,1.96*	**1.58** (1.46,1.64) *1.31,1.96*
	**Right Post Hipp**.	**1.73** (1.51,1.87) *1.46,2.4*	**1.87** (1.52,1.98) *1.39,2.1*	**1.02** (0.98,1.08) *0.89,1.43*	**0.96** (0.98,1.01) *0.84,1.22*	**1.66** (1.53,1.76) *1.3,2.07*	**1.76** (1.6,2.07) *1.5,2.36*
	**Left Post Hipp**.	**1.67** (1.64,1.78) *1.45,1.93*	**1.85** (1.68,2.04) *1.52,2.29*	**0.96** (0.78,0.99) *0.73,1.08*	**0.97** (0.84,1.16) *0.72,1.41*	**1.79** (1.73,1.99) *1.66,2.22*	**1.92** (1.6,2.08) *1.48,2.71*
**Group B**	**Right PFC**	**1.53** (1.34,1.81) *1.10, 1.86*	**1.60** (1.44,1.7) *1.31,1.8*	**1.17** (0.86,1.36) *0.77,1.16*	**1.04** (0.88,1.17) *0.85,1.32*	**1.32** (1.22,1.7) *1.15,2.16*	**1.54** (1.35,1.83) *0.99,1.95*
	**Left PFC**	**1.55** (1.4, 1.68) *1.25,1.75*	**1.65** (1.49,1.77) *1.34,1.91*	**0.98** (0.92,1.04) *0.76,1.13*	**1.03** (0.95,1.09) *0.8,1.20*	**1.47** (1.4,1.73) *1.34,2.28*	**1.59** (1.43,1.79) *1.29,1.97*
	**Midline PFC**	**1.46** (1.36,1.6) *1.33,1.72*	**1.54** (1.24,1.81) *1.2, 1.92*	**0.96** (0.86,1.0) *0.65,1.12*	**0.98** (0.88,1.17) *0.83,1.44*	**1.48** (1.42,1.94) *1.37,2.14*	**1.44** (1.3,1.69) *1.04,2.06*
	**Right Ant. Hipp**.	**1.58** (1.48, 1.7) *1.39,1.92*	**1.67** (1.44,1.81) *1.37,1.94*	**0.98** (0.92,1.08) *0.87,1.23*	**1.03** (0.89,1.11) *0.74,1.36*	**1.60** (1.49,1.73) *1.36,1.88*	**1.72** (1.27,1.77) *1.25,1.89*
	**Left Ant. Hipp**.	**1.50** (1.39,1.62) *1.28,1.87*	**1.65** (1.43,1.75) *1.38,1.92*	**0.98** (0.88,1.08) *0.84,1.21*	**0.98** (0.93,1.11) *0.87,1.37*	**1.54** (1.31,1.72) *1.2,2.1*	**1.64** (1.31,1.84) *1.24,2.21*
	**Right Post Hipp**.	**1.57** (1.51,1.87) *1.21,2.15*	**1.59** (1.45,1.66) *1.18,1.69*	**0.92** (0.88,1.13) *0.71,1.26*	**0.91** (0.77,1.03) *0.75,1.13*	**1.69** (1.64,1.73) *1.54,1.87*	**1.65** (1.52,1.73) *1.49,1.73*
	**Left Post Hipp**.	**1.53** (1.5, 1.72) *1.22,1.83*	**1.49** (1.39,1.69) *1.17,1.92*	**0.90** (0.82,0.99) *0.74,1.14*	**0.92** (0.83,1.1) *0.77,1.19*	**1.68** (1.58,1.96) *1.41,2.07*	**1.61** (1.52,1.72) *1.48,1.73*

Key: Median values are reported in bold, with Lower and Upper Quartiles in parentheses and minimum/maximum values in italics.

### Correlations

Significant correlations were obtained between behavioural measures for TWL and Semantic Clustering on the CVLT (r = +0.51, df = 21, p = 0.019) and between Words Learned and Proactive Interference (r = -0.62, df = 21, p = 0.002). When the groups were analysed separately, these same correlations were again evident for Group A (TWL & Semantic Clustering: r = +0.68, df = 11, p = 0.022; Words Learned & Proactive Interference: r = -0.78, df = 10, p = 0.004), while for Group B a significant correlation was found for Words Learned and Subjective Clustering (r = +0.78, df = 10, p = 0.008).

When behavioural measures were correlated with differences in NAA/(tCr+Cho) ratios at each VOI, significant correlations were found for left PFC and left hippocampus. At the left prefrontal VOI, TWL (r = -0.5, df = 15, p = 0.057), Trial 1 recall (r = -0.54, df = 15, p = 0.036) and Total Recall Trials 1-5 (r = -0.67, df = 15, p = 0.006) elicited negative correlations. At the left anterior hippocampus, TWL (r = -0.52, df = 14, p = 0.058), Short Delay Cued Recall (r = -0.53, df = 14, p = 0.054) and Long Delay Free Recall (r = -0.52, df = 14, p = 0.058) also produced negative correlations. Significant positive correlations were seen at the left posterior hippocampal VOI for Trial 5 recall (r = +0.59, df = 15, p = 0.021) and Semantic Clustering (r = +0.52, df = 15, p = 0.05). In addition, a significant negative correlation was found at the right posterior hippocampal VOI for Proactive Interference (r = - 0.52, df = 14, p = 0.059).

When the analysis was repeated for each group separately, Group A showed correlations at the midline prefrontal voxel for TWL (r = +0.73, df = 9, p = 0.025), and at the left posterior hippocampus for Words Learned (r = -0.71, df = 9, p = 0.032) and Semantic Clustering (r = +0.75, df = 9, p = 0.021). Group B showed correlations at prefrontal VOIs bilaterally and at the right hippocampus. The left PFC produced negative correlations between metabolism change and TWL (r = -0.8, df = 6, p = 0.056), Trial 1 recall (r = -0.83, df = 6, p = 0.042), Total Recall Trials 1-5 (r = -0.92, df = 6, p = 0.009) and Subjective Clustering (r = -0.9, df = 6, p = 0.014). The right prefrontal cortex showed positive correlations between metabolism and Trial 1 recall (r = +0.87, df = 6, p = 0.026) and Subjective Clustering (r = +0.88, df = 6, p = 0.014). There was a positive correlation at the right anterior hippocampus for Short Delay Cued Recall (r = +0.89, df = 5, p = 0.044), and at the right posterior hippocampus for TWL (r = -0.86, df = 6, p = 0.027).

## Discussion

In this study, two groups of normal older adults were trained with prolonged rote learning over six weeks, with memory performance and brain metabolism measures assessed pre- and post-training. We predicted that such prolonged learning would, by virtue of the repeated activation of memory structures, bring about performance benefits on behavioural memory measures, and that these effects would be confined to verbal-based tests. We found that this training regime did produce an enhancement in memory function in the compliant training Group A, but not in the non-compliant training Group B. These memory gains were specific to three independent verbally-based measures (RBMT, CVLT and MMQ), and could not be attributed to extraneous factors such as anxiety, depression or attention. The absence of such an effect in Group B suggests that compliance with weekly learning was the crucial determinant of this facilitation. In addition, Group A displayed a metabolic change relative to Group B at Week 12 in the left posterior hippocampus, implying that this verbal memory enhancement may have a hippocampal substrate.

It appears that Group A expended greater effort in learning than Group B, as evidenced by Group A's larger mean increase in weekly words learned. By contrast, Group B participants showed little improvement in weekly learning; five participants in this group began at a high level of weekly learning (>400 words/week), and maintained this throughout the six weeks, while another four participants maintained a low weekly word total from start to finish (<400 words/week). This lack of adequate (or effortful) compliance with weekly rote learning may therefore explain the lack of a delayed verbal/episodic memory enhancement in this group. It may be that a minimum threshold of repetitive activation is required in key brain structures if the benefits of prolonged activation and usage are to be observed.

A delayed memory enhancement was found for Group A six weeks after the end of their weekly rote learning regimen, and this enhancement was observed for three independent verbal/episodic memory measures: immediate recall on the RBMT short story, four sub-measures of the CVLT, and recall of everyday events on the MMQ. No such improvement was found for the visuo-spatial task, or any of the control measures employed, and can therefore not be explained by generalised increases in arousal or attention, fluctuations in depression or anxiety, or other confounding variables. These effects may be thus attributable to the prolonged period of rote rehearsal of verbal material. This verbally-mediated memory gain emerged six weeks after a period of no learning. This suggests that the learning benefits accrued by sustained rote rehearsal could require a latent period before they emerge. The duration of this latent period is unknown - it is possible that the six-week follow-up used in the present study revealed these memory gains at their greatest magnitude, but it is equally possible that the data collected at this time point represent a position on either an upward or downward curve of facilitation. Further study will be required to determine the exact time course of this delayed facilitation effect. Irrespective of this, the fact that these effects were observed on a range of laboratory measures and the recall of everyday real-life events demonstrates that this is a highly robust effect, and appears specific to the verbal/episodic memory domain.

The behavioural memory improvement was accompanied by a metabolic difference between groups difference at only one voxel of interest (VOI), the left posterior hippocampus. The left hippocampal region has been reliably associated with verbal and episodic memory performance in normal humans using imaging, and in lesioned patients (see [5]). As such, it is not unreasonable to attribute the behavioural effects to alterations in the metabolic activity of this region as a result of the rote learning regime. A comparable effect was reported in Valenzuela et al. [30], where a five week training period with a spatially-based strategy produced memory enhancement and changes in metabolite levels in right hippocampus, however they reported an unexplained reduction in NAA/Cr measures in the hippocampus following memory training. The present results may represent evidence of a complimentary process for verbal learning, subserved by the left hippocampal formation. At present we can only hypothesise as to the mechanism of action of this process; however, the repeated activation of brain structures associated with rote learning, particularly the projection from left PFC to hippocampus, may have led to increases in neuronal viability and health in these regions, as indexed by increased NAA/(Cho+tCr) ratio. These benefits may be slow to emerge, but have the effect of facilitating future learning of new information, in a manner akin to the elevated memory performance of the mentally-active nuns in Snowdon's studies [21-23]. Furthermore, Riby et al. [49] recently demonstrated that glucose ingestion facilitated episodic memory performance in a sample of healthy elderly participants. Prolonged learning with its concomitant increased bloodflow to memory structures may have had a comparable glucose-mediated effect. The exact neurometabolic mechanism behind this effect notwithstanding, it can be cautiously asserted that an extended period of rote rehearsal leads to enhanced future learning accompanied by changes in the markers of cell health in a key memory structure of the brain.

When changes in metabolism for Week 6 to Week 12 were correlated with changes in behavioural measures, significant correlations tended to cluster at specific VOIs. When the groups were collapsed, two regions, left PFC and left hippocampus (anterior and posterior) were associated with changes in seven of the eight measures tested. This is further support for the suggestion that, on the whole, the well-established anatomical connection between left PFC and left hippocampal formation (the uncinate fascicle; [29]) may have been activated in this study. An anatomical dissociation was evident when the groups were considered separately:

Group A's behavioural measures were correlated with changes primarily in left posterior hippocampus, while for Group B left and right PFC were most strongly implicated.

Due to limitations of the MR system employed in this study the minimal VOI available was 8 cm^3^. Thus the hippocampal VOIs also included extrahippocampal tissues such as the amygdale, the entorhinal cortex, the parahippocampal gyrus and CSF in the temporal horn of the lateral ventricle in keeping with previous studies employing single-voxel MRS [50-56]. In a previous study utilising single voxel proton MRS to evaluate hippocampal sclerosis, Chang et al. [51] utilised small and large VOIs and discussed the partial volume effects of the larger voxels and the inadequate signal-to-noise ratio of smaller voxels resulting in inadequate spectra. For the current study special care was taken when positioning the VOIs to ensure maximal hippocampal coverage, to minimise partial volume effects from other tissues, to minimise potential magnetic susceptibility artifacts from the skull base and sphenoid sinus, and to minimise potential spectral contamination from fat in the skull base. The use of two-dimensional chemical shift imaging, which allows improved sampling of smaller VOIs, the measurement of the absolute metabolite levels, the use of within voxel tissue segmentation, and higher field strength magnets would improve the MRS data in future studies as the current approach was somewhat limited by the technology available [51,56]. However Waldman and Rai [55] suggest that the determination of metabolite ratios rather than absolute concentrations circumvents the necessity for segmentation to correct for CSF in the acquisition voxel and that such processes inevitably introduce errors.

Although these data, along with those of previous studies, suggest some promise for MRS in the evaluation of memory it must be noted that the relatively small sample size is a limitation and extrapolation of observations requires caution. Further limitations include the lack of a true comparison group and the curtailment of the age range. Strengths included the recruitment of a diverse sample of community based older participants which reduces the possibility of sampling bias and the use of a detailed and standardised neuropsychological protocol.

## Conclusion

In conclusion, we have here demonstrated that, when compliance is high, a prolonged period of repetitive rote learning may lead to improvements in verbal/episodic memory which emerge in the weeks following learning cessation, and that these benefits are not confined to laboratory-based indices of memory. Furthermore, these benefits appear to be associated with metabolic changes in the left posterior hippocampus, which may reveal the health implications for key brain structures of repetitive activation and regular usage in healthy ageing.

## Authors' contributions

RAPR carried out behavioural testing and participant recruitment, analysed the behavioural data, plotted the figures and wrote the manuscript. JMcN carried out participant scanning, assisted with data analysis, and contributed to writing of manuscript; SLM and JH carried out behavioural testing and participant recruitment, and contributed to analysis. PB, CPD, MF and DMcM facilitated access to and testing with the MR scanner; JP carried out participant scanning and assisted with data analysis. SS and MAM provided specific test batteries during participant testing and assisted with data analysis and interpretation; IHR and SOM provided the initial impetus for the study and oversaw the execution of the study. All authors have read and approved the final manuscript, with the exception of DMcM, who passed away in 2006.
